# Development and feasibility of a virtual reality-based exergaming program to enhance cardiopulmonary fitness in children with developmental coordination disorder

**DOI:** 10.3389/fped.2023.1238471

**Published:** 2023-12-20

**Authors:** Ya-Ju Ju, Yi-Chun Du, Hsien-Chun Huang, Po-Sen Hu Kao, Rong-Ju Cherng

**Affiliations:** ^1^ Institute of Allied Health Sciences, College of Medicine, National Cheng Kung University, Tainan, Taiwan; ^2^ Department of Biomedical Engineering, College of Engineering, National Cheng Kung University, Tainan, Taiwan; ^3^ Medical Device Innovation Center, National Cheng Kung University, Tainan, Taiwan; ^4^ Department of Product Engineering Division, Voltafield Technology Corporation, Hsinchu, Taiwan; ^5^ Department of Physical Medicine and Rehabilitation, Landseed International Hospital, Taoyuan, Taiwan; ^6^ Department of Physical Therapy, College of Medicine, National Cheng Kung University, Tainan, Taiwan

**Keywords:** developmental coordination disorder, cardiopulmonary fitness, exergame, virtual reality, motor skill deficit

## Abstract

**Introduction:**

Developmental coordination disorder (DCD) is a neurodevelopmental disorder characterized by motor skill deficits. Such deficits often limit children's participation in physical activities, further affecting their overall health, including through reduced cardiopulmonary fitness. Because virtual reality (VR) devices offer interactive games and activities that require various movements and coordination, they can serve as motivating and enjoyable means for children to perform physical exercise. In this study, we developed a VR-based exergaming system and tested its ability to enhance the cardiopulmonary fitness of children with DCD.

**Materials and methods:**

A total of 13 children with DCD and 10 young adults were recruited in phase I to examine the test–retest reliability and concurrent validity of our system (including a custom-made heart rate monitor) with a commercial heart rate device. In phase II, we included an additional 13 children with DCD to test the feasibility of the system. We tested the outcomes using the enjoyment rating scale, intrinsic motivation inventory (IMI), and 20-m shuttle run test (20mSRT).

**Results:**

In phase I, test–retest reliability was good to excellent in the static task and moderate to good in the dynamic task. Concurrent validity was excellent in both tasks. In phase II, more than half of the children (18 out of 26) assigned the maximum rating for their enjoyment of the game; they also had high average scores on the IMI. Furthermore, after the 8-week training using the VR program, the average running distance of the 26 children in the 20mSRT had increased significantly from 129.23 m to 176.92 m (*p* < 0.001).

**Conclusion:**

Our VR-based exergaming program can serve as an alternative intervention for enhancing cardiopulmonary fitness in children with DCD.

## 1. Introduction

Developmental coordination disorder (DCD) is a neurodevelopmental disorder characterized by motor skill deficits. These deficits experienced by individuals with DCD often limit their performance of and participation in physical activities, further harming their overall health through, for example, reduced cardiopulmonary fitness. A systematic review indicated that the oxygen consumption at peak physical exertion (⩒O_2max_) of typical children and adolescents aged 8–19 years is 35–47 ml/kg/min during cardiopulmonary endurance exercise (1). However, the ⩒O_2max_ in children with DCD is much lower, only 33.5–34.4 ml/kg/min on average (2). In addition, a longitudinal study revealed that the difference in cardiopulmonary endurance between children with DCD and typical children increased with age (3).

The American College of Sports Medicine indicates that cardiopulmonary fitness is promoted by aerobic exercise, especially exercise that increases the heart rate to 65%–75% of its maximum. Aerobic exercise can improve cardiopulmonary fitness, muscle power, body composition, cognition, and inhibitory control (4–8). Lau et al. also demonstrated that aerobic exercise and moderate-to-vigorous physical activity from 19.2 to 29.33 ml/kg/min could improve cardiopulmonary fitness through increased ⩒O_2max_ (9). Although studies have demonstrated the benefits of traditional aerobic exercise, children often regard this exercise as boring or difficult.

In virtual reality (VR), multimedia technology and an interface between the human body and a computer enable people to experience a virtual environment and the objects in it as they do in the real world. Because VR offers interactive games and activities requiring various movements and coordination, it represents a motivating and enjoyable approach to physical exercise. VR technology is thus highly suitable for combining exercise with gaming (exergaming). Studies have indicated that individuals using VR training outperformed those using traditional training programs in the Fugl–Meyer assessment, Berg balance scale, timed up-and-go test, functional reach test, and 10-m walking test (10–12). Moreover, individuals with cerebral palsy or Down syndrome exhibited marked improvements in their motor coordination, balance, and ambulatory function after undergoing weekly 1-h VR training sessions, highlighting the efficacy of this training for improving physical capabilities (13, 14). Many scholars have reported improved motor control in patients undergoing VR training, but the results have been inconsistent. Moreover, studies have yet to examine the effect of VR training in children with DCD.

## 2. Method

### 2.1. Participants

A total of 10 young adults (age: 25.9 ± 4.3 years; 3 men and 7 women) and 13 school-age children with DCD (average age: 8.8 ± 0.9 years; 7 boys and 6 girls) participated in our phase I study to examine the test–retest reliability and concurrent validity of the system and program that we developed with a customer. In our phase II feasibility study, a further 13 children with DCD joined the original 13 children with DCD, leading to a total of 26 participants (age: 8.3 ± 1.0 years, 16 boys and 10 girls). These children completed an 8-week program with our VR device. The young adults were recruited from a university for convenience. Individuals with a neurological, musculoskeletal, or cardiopulmonary condition were excluded. Children with DCD were screened and referred by teachers from local primary schools. DCD diagnosis was based on the criteria of the *Diagnostic and Statistical Manual of Mental Disorders, Fifth Edition*. We used the Movement Assessment Battery for Children, Second Edition (MABC-2) to confirm the children's motor skill deficits. Motor difficulty was indicated by a total score at or below the 16th percentile of the age norm. This study was approved by the ethics committee of our university, and the participants provided informed consent prior to their participation, in accordance with the Declaration of Helsinki.

Table 1 presents the demographic characteristics of the participants. All of the children with DCD in the phase I and II studies had total MABC-2 scores that were below the 16th percentile. Their subtest scores also revealed motor difficulties in aiming, catching, and balance.

**Table 1:** The demographic characteristics of phase I and II participants.

	pDCD children	pDCD children	Adult
Number	26	13	10
Gender (Male/Female)	16/10	7/6	3/7
Age (Years)	8.3 ± 0.99	8.8 ± 0.89	25.9 ± 4.28
Height (cm)	128.67 ± 8.9	131.71 ± 8.62	166.3 ± 6.38
Weight (kg)	31.83 ± 8.61	34.07 ± 9.86	57.5 ± 10.07
Total score of C-PPVT-R (%)	82.62 ± 15.92	88.3 ± 7.88	
Total score of MABC-2 (%)	6.69 ± 3.3	7.54 ± 3.73	
Manual Dexterity (%)	28.96 ± 16.87	28.31 ± 16.03	
Aiming and Catching (%)	13.35 ± 14.88	16.24 ± 13.74	
Balance (%)	9.46 ± 8.41	9.69 ± 7.73	

### 2.2. Equipment and apparatus

#### 2.2.1. Hardware

ProComp5 Infiniti encoder (SA7525, Thought Technology Ltd., 2007, Canada.)

The ProComp5 Infiniti™ (SA7525, Thought Technology Ltd., 2007, Canada.) is encased in an ergonomically designed housing, requiring only a USB port for seamless connectivity to the computer. With two sensor channels, it ensures optimal signal fidelity, providing a high sampling rate of 2,048 samples per second for observing raw heart rate signals.

##### 2.2.1.1. VIVE pro (HTC Corporation, Taiwan)

**Figure 1:**
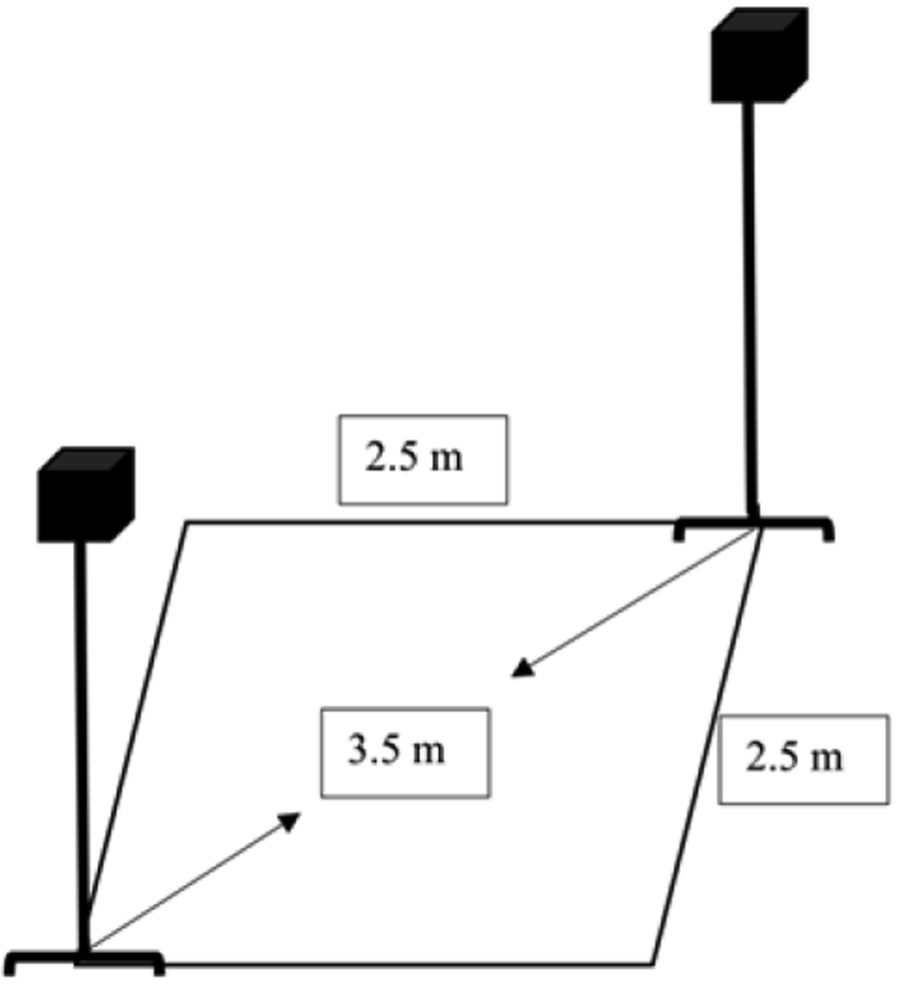
The experiment setting. In the virtual reality environment setup, cameras are placed at diagonal corners with a spacing of 3.5 m, forming a rectangular frame measuring 2.5 m in length and width. Children will perform action training within this area.

To develop our VR-based exergaming program, we used the VIVE pro (HTC Corporation, Taiwan) as our application programming interface. The VIVE pro is a portable and convenient VR system that includes a head monitor and four sensors, two each for the hands and feet. The head monitor has a 110° field of view, a resolution of 1,080 × 1,200 pixels per eye, and a refresh rate of 90 Hz. We added a custom-made heart rate monitor to this system. The sensor was attached to the pulp of the index finger of the non-dominant hand and fixed at the wrist by a wire. Figure 1 demonstrates the experimental setting.

#### 2.2.2. Software

**Figure 2:**
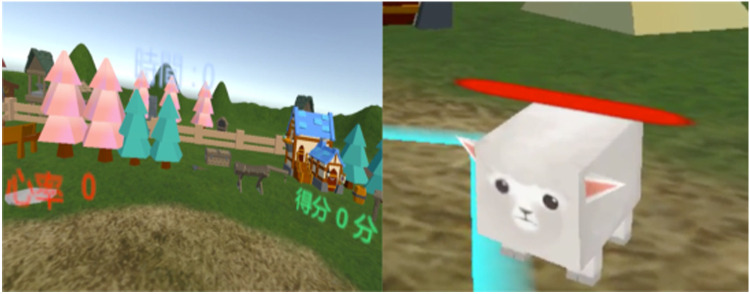
The VR exergaming program. The virtual reality system creates a cardiorespiratory fitness exercise game with the background of small animals escaping from a farm. Children are required to help the farmer catch the small animals and bring them back to the farm.

We designed an exergaming program named “Animal Escape” by using Unity software (Unity 5.0, Unity-Technologies, 2004, San Francisco, California, USA; Figure 2). The game content was task oriented, with a focus on repetitive practice of real-life functional tasks.

Consistent with the principles of aerobic exercise training, the game is structured as follows: first, a 5-min warm-up stretching activity, then 20 min of training activities, and finally, a 5-min cool-down activity (15). The game involves returning escaped animals to their farm. Different animals appear one after another and hide in random locations. As soon as one appears, the player must catch it. The interval between the appearance of animals is 1 s during the warm-up and cool-down stages but 0.5 s or 0.25 s during the training stage. Players receive a coin reward after having caught 100 animals.

#### 2.2.3. Movement assessment battery for children-second edition (MABC-2)

The MABC-2 is a norm-referenced motor test for children aged 3–16 years (16).

The test is divided into three age bands (3–6, 7–10, and 11–16 years) and consists of three subtests (manual dexterity, aiming and catching, and balance). The total score on the three subtests is used to diagnose motor difficulties in children. A total score at or below the 5th percentile denotes a significant movement difficulty, whereas scores between the 5th and 15th percentiles indicate the child is at risk of having movement difficulties (16). The MABC-2 test has been widely used in hospitals and research because of its good-to-excellent test–retest reliability and validity (17). In this study, children with a total MABC-2 score lower than the 16th percentile were regarded as having DCD.

### 2.3. Outcome measurement

#### 2.3.1. Enjoyment rating scale

After the whole training session, we used the enjoyment rating scale (Table 2) to measure how much the children enjoyed the exergaming program. We used a 5-point Likert scale (0: no fun at all, 1: boring, 2: a bit of fun, 3: fun, and 4: super fun) (18, 19).

**Table 2:** The results of the enjoyment scale.

Enjoyment Scale	pDCD children (*N* = 26)
0 (no fun at all)	0
1 (boring)	1
2 (a bit of fun)	2
3 (fun)	5
4 (super fun)	18

#### 2.3.2. Intrinsic motivation inventory scale (IMI scale)

The intrinsic motivation inventory (IMI), a multidimensional measurement device that is grounded in self-determination theory, is intended to assess participants' subjective experiences related to a target activity. There are seven subscales of IMI. However, we only used the following three subscales of the IMI: interest/enjoyment (seven items), perceived competence (six items), and effort/importance (six items; Table 3). Each item is scored from 1 to 7 points, with more points indicating more favorable performance. The IMI has been applied in healthy adults, athletes, patients with schizophrenia, and patients with stroke (20–23). This scale has favorable test–retest reliability (intraclass correlation *r* = 0.77) (20).

**Table 3:** The results of the IMI score.

Part I. Interesting condition	Average ± SD
1. I enjoyed doing the game	6.42 ± 1.5
2. I liked the virtual reality game; it was better than the traditional exercise.	6.73 ± 0.72
3. The virtual reality game was fun to do.	6.46 ± 1.47
4. I thought the game was quite enjoyable	6.15 ± 1.71
5. While I was doing the game, I was thinking about how much I enjoyed it.	6.11 ± 1.7
6. I thought the game was a boring activity.	1.26 ± 1.18
7. The game did not hold my attention at all.	1.96 ± 2.04
Scale of Interesting condition (Average ± SD)	6.37 ± 1.42
Part II. Performance condition	
Perceived competence	
1. I think I am pretty good at the game.	6 ± 1.9
2. I think I did pretty well at the game compared to other children.	5.04 ± 2.4
3. After working at the game for a while, I felt I could do it.	5.8 ± 2
4. I am satisfied with my performance in the game.	6.24 ± 1.23
5. I am good at the game.	5.75 ± 1.89
6. I think I am not good at the game.	1.48 ± 1.29
Average ± SD of the clockwise items	5.76 ± 0.44
Effort/importance	
1. I put a lot of my attention into the game.	6.28 ± 1.17
2. I tried very hard in the game.	6.4 ± 1.22
3. It was important to me to do the game.	4.76 ± 2.5
4. I could not do the game very well.	1.48 ± 1.29
5. I did not try very hard in the game.	1.52 ± 1.44
6. I did not put a lot of my attention into the game.	1.83 ± 1.88
Average ± SD of the clockwise items	5.81 ± 0.91
Scale of performance condition of the clockwise items (average ± SD)	5.78 ± 0.32
Total of score (average ± SD)	6.07 ± 0.87

#### 2.3.3. Twenty-meter shuttle run test

The 20-m shuttle run test (20mSRT) is a common field test for measuring cardiorespiratory fitness and has favorable test–retest reliability (*r* = 0.89) (24, 25). In the test, a participant must run back and forth between two lines before a beep sounds. The test ends when the child fails to reach the line before the designated time. The total distance ran represents the test performance.

### 2.4. Procedure

A total of 10 young adults and 13 children with DCD participated in phase I of our study, in which we examined the test–retest and concurrent validity of our custom-designed heart rate device. We measured the participants' heart rate under static and dynamic conditions. In the static condition, a participant was asked to sit quietly and relax on a chair with its height adjusted to their leg length to ensure hip and knee flexion of 90° and that their feet were flat on the floor. The participant's hands were naturally placed on their thighs. The examiner twice recorded the participant's heart rate for 1 min, with the interval between measurements being 30 min. Subsequently, for the dynamic task, the participant performed a modified stepping test; the adults and children used 35 cm and 20 cm steps, respectively. The task involved 24 up-and-down cycles per minute for 2 min, yielding 96 steps. Immediately after the test, the participant sat on a chair in the same manner as previously described, and their heart rate was measured for 1 min; the measurement was repeated after 30 min. In our phase II study for measuring the feasibility of the system, another 13 children with DCD joined the original 13 children for an 8-week exergaming program using our VR device. After the 8-week program, all participants completed the enjoyment rating scale, IMI, and 20mSRT (26). All participants completed the enjoyment rating scale and IMI after the physical fitness game.

### 2.5. Statistical analysis

The participants' demographic data and their scores on the enjoyment rating scale and IMI are presented as means and standard deviations. We examined the test–retest reliability and concurrent validity of our device by using Pearson correlation analysis (i.e., calculating the intraclass correlation coefficient, ICC; SPSS version 20; Chicago, IL, USA). Pearson correlation coefficients of <0.5, 0.5–0.75, and >0.75 indicate no correlation, moderate-to-good correlation, and excellent correlation, respectively.

## 3. Results

### 3.1. The test–retest reliability and concurrent validity with self-developed heart rate monitor device

In the static task, the device exhibited good-to-excellent test–retest reliability (adult group ICC = 0.946, *p *= 0.000; children group ICC = 0.768, *p *= 0.001) and concurrent validity (adult group *r* = 0.992, *p *= .000; children group *r* = 0.943, *p *= .000). In the dynamic task, the device exhibited moderate-to-good test–retest reliability (adult group ICC = 0.93, *p *= 0.000; children group ICC = 0.913, *p *= 0.000) and excellent concurrent validity (adult group *r* = 0.98, *p *= .000; children group *r* = 0.967, *p *= .000).

### 3.2. Feasibility of the VR-based training system

The average rating on the enjoyment rating scale was 3.6 ± 0.8 points (maximum = 4; Table 2). In terms of the IMI, the average scores were 6.4 ± 1.4 and 5.8 ± 0.3 points for the interest and performance subdimensions, respectively (Table 3). After 8 weeks of training with the VR-based exergame, the children with DCD exhibited a significant improvement in the distance they ran in the 20mSRT from 129.23 m to 176.92 m (*p *< 0.001). However, their ⩒O_2max_ was not significantly better after the training program.

## 4. Discussion

In this study, we examined the test–retest reliability, concurrent validity, and feasibility of an exergame system aimed at improving cardiopulmonary fitness. In the feasibility test, we considered the participants' enjoyment, intrinsic motivation, and performance on the 20mSRT after 8 weeks of training. Our results indicated that the concurrent validity of the heart rate device was good to excellent and that the participants enjoyed using the system. They also had high scores for the interest and performance subdimensions of the IMI.

### 4.1. High reliability and validity of the heart rate device

Our heart rate device exhibited good-to-excellent test–retest reliability and concurrent validity. Individuals with gross motor deficits have consistently been found to have lower cardiopulmonary fitness and reduced anaerobic exercise capacity (27, 28). Cardiopulmonary fitness is commonly assessed using heart rate and ⩒O_2max_. In our study, the average resting heart rate in the healthy adults was 80–83 beats per min, consistent with findings from previous studies (29, 30). In addition, the children with DCD in our sample had higher resting heart rates than typically developing children, ranging from 90 to 97 beats per min. This can be attributed to their engagement in static activities for prolonged periods and limited participation in moderate-to-vigorous-intensity exercise (31). We employed static and dynamic tasks to observe changes in heart rate. During the dynamic task, our heart rate device exhibited slightly lower test–retest reliability than a commercial heart rate device with cable transmission. However, our heart rate device remains viable because of its portability and wireless transmission capability (contingent on a stable wireless connection).

### 4.2. Change in fitness level after VR training

#### 4.2.1. Objective change

The 20mSRT is a widely recognized measurement tool for assessing ⩒O_2max_ in children and adults. Numerous studies have reported that children with DCD tend to cover a shorter distance during the 20mSRT than do typically developing children, indicating lower cardiorespiratory fitness in children with DCD (28). Furthermore, the 20mSRT has been proven effective in detecting changes in fitness following training interventions. After our participants used our VR fitness program, the distance they covered in the 20mSRT was significantly higher than that before. However, we discovered no significant change in their ⩒O_2max_. The VR game helped improve the participants' cardiopulmonary fitness because they could run longer distances than before playing the game. Other research has also demonstrated that physical fitness programs can improve muscle power and endurance (32). Leger et al. reported that for estimating the ⩒O_2max_ of children aged 6–18 years, data from the multistage 20mSRT had good reliability. Additionally, relative to a treadmill test, the 20mSRT was discovered to have moderate reliability for estimating ⩒O_2max_ in children aged 6–10 years (33). Although the estimated ⩒O_2max_ derived from the multistage 20mSRT is a useful option for estimating cardiorespiratory fitness, it may yield more accurate results in adults than in children (34). Notably, discrepancies may have arisen between the distance covered during the 20mSRT and the estimated ⩒O_2max_, potentially leading to inconsistent performance rankings in our study. We conducted the test outdoors to provide a natural environment for the children. Additionally, some of the children may have felt hot and tired during the outdoor testing, and these factors may have affected their motivation and performance.

#### 4.2.2. Perceived change

We used the enjoyment rating scale, which has favorable reliability (35, 36), to measure the children's motivation. The children in our study comprehended the scale's meaning and provided responses accordingly. Most of the children reported having fun while playing our game. However, three children expressed boredom or less enjoyment and perceived the game as too easy, leading to low motivation. To assess our participants' abilities, performance, and mental engagement, we used the IMI (37). The children understood the scale items and could thus accurately answer them. The children's answers on the IMI prior to playing the game indicated that most of them had high expectations. Moreover, we observed that the children concentrated and exerted considerable effort during the game because they believed that maintaining their motivation was crucial given the perceived difficulty level. After playing the game, most of the children reported liking it and had increased confidence because their abilities and physical fitness had improved. Additionally, the VR game fostered closer relationships between the children and their classmates.

### 4.3. Study limitation

Because of the unstable wireless connection of the heart rate device during the dynamic task, only moderate test–retest reliability was discovered for this task. Moreover, the estimated ⩒O_2max_ of our 26 participants, measured using the multistage 20mSRT, was not improved after our VR training program. We recognize that this test may not be the most accurate for measuring ⩒O_2max_ changes, but it was the only one we employed. Three children provided low enjoyment scores, suggesting room for improvement in the game's content to make it more challenging and interesting. In addition, the small sample size affects the study's generalizability and application.

### 4.4. Future study

We aim to adopt a higher-quality wireless transmission module to improve our heart rate device performance. To enhance the children's enjoyment of the game, we plan to introduce different game levels to challenge the children appropriately in accordance with their abilities.

## 5. Conclusion

Our device has good reliability and validity. Additionally, we obtained preliminary evidence that our VR game can improve physical fitness, but further research with a larger sample size is necessary. The VR game and heart rate device that we have designed may be useful for physical fitness interventions in the future.

## Data Availability

The original contributions presented in the study are included in the article/Supplementary Material, further inquiries can be directed to the corresponding author.
